# Evaluating Infrared Absorption Parameters for Low-Temperature Ices Using Reflection–Absorption Infrared Spectroscopy

**DOI:** 10.1021/acsearthspacechem.4c00394

**Published:** 2025-03-04

**Authors:** Jack E. Fulker, Martin McCoustra, Wendy A. Brown

**Affiliations:** 1 Department of Chemistry, University of Sussex, Falmer, Brighton BN1 9QJ, U.K.; 2 Institute of Chemical Sciences, Heriot-Watt University, Edinburgh EH14 4AS, U.K.

**Keywords:** infrared spectroscopy, absorption coefficient, absorption cross-section, absorption band strength, graphite, metal-surface selection rule, metal-surface enhancement factor

## Abstract

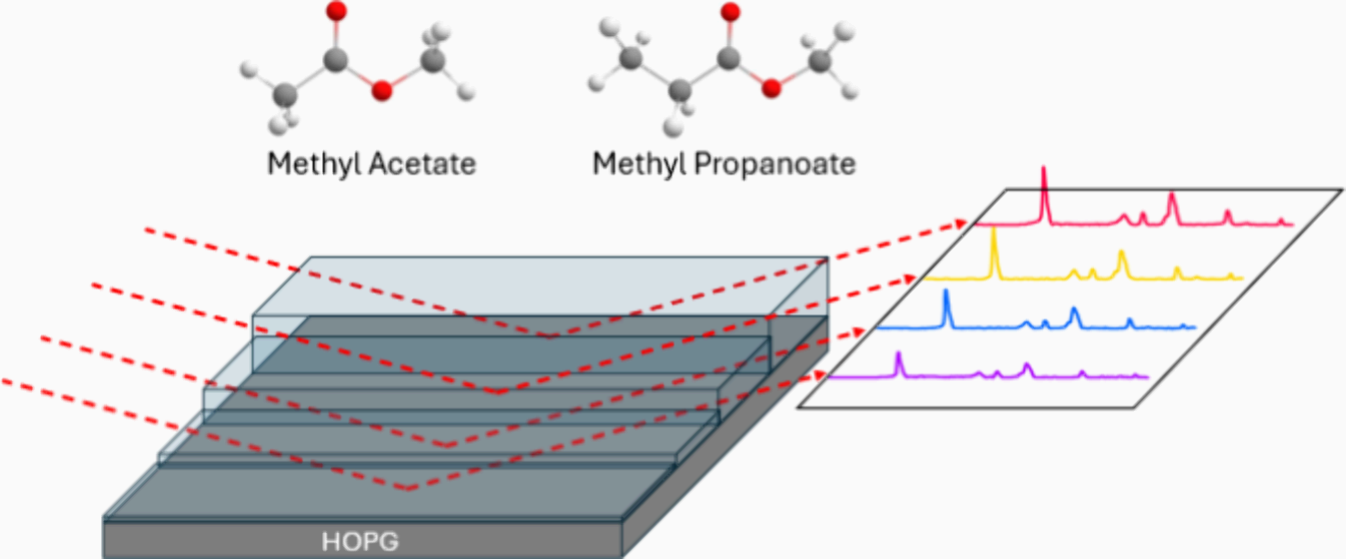

We present infrared absorption coefficients, cross sections, and band strengths calculated from reflection–absorption infrared spectroscopy (RAIRS) data of molecular ices deposited on a highly oriented pyrolytic graphite (HOPG) surface at 28 K. We also describe an estimated conversion factor (encompassing considerations from the metal-surface selection rule and metal-surface enhancement factor for graphite) to explain how these parameters compare to those calculated using transmission infrared methods. Further, we discuss the limitations of this method and the assumptions which must be considered when evaluating absorption parameters from RAIRS data. The two species studied here (methyl acetate and methyl propanoate) were chosen to test these methods, as they are both astrochemically relevant and have previously been reported in the literature, providing a reliable benchmark.

## Introduction

Infrared (IR) spectroscopy is an important technique used across almost every field of chemistry as both a fingerprinting tool for molecular structure and as a method for following molecular dynamics and chemical processes.^1^ It is also particularly important in astrochemistry, providing insight into the composition and physical conditions of interstellar and circumstellar environments through direct observational spectroscopy of planetary atmospheres and molecular clouds.^2−4^ IR spectroscopy is particularly well-suited for studying molecular ices such as those found on dust grains in the interstellar medium (ISM), which are integral to astrochemical processes and the evolution of complex organic molecules (COMs).^5−9^ As infrared radiation can penetrate through dense clouds that obscure other wavelengths, IR spectroscopy enables observations in otherwise inaccessible regions, providing essential data for modeling the chemical pathways leading from simple interstellar species to prebiotic molecules.^10,11^ IR spectroscopy therefore provides a unique window into the molecular complexity of the ISM and is fundamental to understanding the chemical evolution of these environments. This is clearly demonstrated by the James Webb Space Telescope (JWST) IR capabilities, with COMs such as simple alcohols, esters and amines among many others already being detected in molecular clouds and protostellar systems.^2,12,13^

While the interpretation of IR spectra can be straightforward for well-understood systems such as those in carefully controlled laboratory settings, interpreting the spectra collected in astrochemical environments requires a deeper understanding of what defines the intensity and shape of absorption bands. IR absorption coefficients (α’), cross sections (σ), and band strengths (Α’) are parameters that describe how molecules absorb IR radiation at specific wavelengths, defining their chemical structure and vibrational modes.^1,14,15^ The IR absorption coefficient quantifies the absorbance of a species per unit concentration and path length, which provides a measure of how effectively a molecule absorbs IR radiation as a function of the light’s wavelength.^1^ The IR absorption cross-section is a molecular property that indicates the probability of an IR photon being absorbed by a single molecule, which is key in both gas- and solid-phase studies, where molecular density and cross sections together influence the observed absorption signal.^1^ IR band strengths, sometimes referred to as integrated absorption cross sections, represent the total absorption of a particular vibrational mode across a specified frequency range and are measured as the integral of the absorption cross-section over the whole band.^1^ Band strengths therefore account for the convolution of both the wavelength dependency of absorption cross sections across an absorption band and the instrumental response of the spectrometer used to record the data. Band strengths are particularly important in quantifying molecular abundances, as they link observed IR spectra to the concentration of specific molecular species, even in complex mixtures. These parameters are therefore essential in observational astrochemistry for translating astronomical IR spectra into quantitative molecular abundances and for characterizing chemical compositions of these environments.^2^

By analyzing the absorption bands from observational spectra in combination with calibrated IR absorption parameters from experimental laboratory studies, the column densities of individual molecular species can be calculated, both in the gas phase and on icy dust grains.^2^ These quantitative measurements may then be used in theoretical models to predict molecular formation and reaction pathways in environments such as dense molecular clouds and protoplanetary disks.^11^

Laboratory studies of these IR parameters are therefore essential in deciphering the results of observational studies. Most molecular ice studies are performed on transmitting surfaces and use transmission IR spectroscopy as a tool to calculate these parameters, since the absorption path length relates directly to the thickness of the molecular ices.^14−21^ However, transmitting surfaces such as KBr do not meet the requirements for many other surface science studies (electron irradiation, low energy electron diffraction (LEED), *etc.*), and so opaque yet reflective (metal or semimetal) surfaces are used instead.^22,23^ On metal and semimetal surfaces, reflection–absorption infrared spectroscopy (RAIRS) can be employed to record IR spectra as reflectance spectra.^24−28^

Currently, very little literature exists concerning the use of RAIRS data to calculate IR absorption parameters. The work of Santos et al. details the calculation of IR absorption band strengths using RAIRS and describes a transmission-to-reflection factor of 3.2 when converting IR absorption band strengths.^29,30^ However, the authors note that this factor applies only to their experimental setup, as it was determined by calibration experiments using a laser interference technique.

There is therefore a need to discuss how parameters calculated using RAIRS compare to those derived from transmission IR experiments, with a focus on the assumptions and limitations of the technique. This work aims to demonstrate how IR absorption coefficients, cross sections, and band strengths can be calculated from RAIRS data recorded for molecular ices grown on a cryogenically cooled graphite surface, using two astrochemically relevant species (methyl acetate (CH_3_COOCH_3_), identified in the Orion nebula,^31^ and methyl propanoate (CH_3_CH_2_COOCH_3_), a likely candidate for future detection^16^) as examples. These species were also chosen due to their relation to another widely observed and studied astrochemical molecule, methyl formate, which has been theorized to play a key role in the chemical evolution of interstellar molecular clouds, cometary ices and protoplanetary systems.^7,9,32,33^ In this work, we outline the methods used to calculate these parameters and the situations under which RAIRS can (and cannot) be used to determine these optical parameters.

## Methodology

Experiments were performed in an ultrahigh vacuum (UHV) chamber with a base pressure of ≤2 × 10^–10^ mbar. The molecular ices were grown via vapor deposition onto a highly oriented pyrolytic graphite (HOPG) surface which was cryogenically cooled to 28 K using a closed-cycle helium refrigerator (SHI-APD). The HOPG surface (Goodfellow Cambridge Ltd.) was cleaned via Scotch tape exfoliation prior to installation,^34^ and then annealed to 250 K in between experiments to remove surface impurities and volatiles. The sample cleanness was confirmed by the absence of any desorption products during annealing, monitored using quadrupole mass spectrometry (QMS, Hiden HAL301/PIC). The temperature of the surface (and by extension the model ices) was monitored using an N-type (nicrosil-nisil) thermocouple sandwiched between the HOPG and coldfinger assembly.

The surface densities, *N*_*m,s*_, of the ices deposited on the HOPG substrate were calculated according to the impingement rate of molecules on the surface over the course of a controlled background gas exposure.
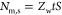
1where *Z*_*w*_ is the wall collision rate, *t* is the exposure time in seconds, and *S* is the sticking coefficient (assumed to be one at cryogenic temperatures). *Z*_*w*_ was calculated as follows,
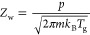
2where *p* is the partial pressure of the adsorbate, *m* is the mass of one molecule of the adsorbate, *k*_*B*_ is the Boltzmann constant, and *T*_*g*_ is the gas temperature. There is also an ion gauge sensitivity factor that is included for the species tested in this work (3.05 and 3.22 for methyl acetate and methyl propanoate respectively).^35^

The thicknesses of the ices, *h*, were then calculated using the molecular densities, ρ, of amorphous methyl acetate and methyl propanoate ices deposited under similar conditions (reported by Hudson et al. as 0.832 g cm^–3^ and 0.702 g cm^–3^ respectively),^14,16^
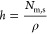
3

The uncertainty in the ice thickness between experiments was checked by comparing temperature-programmed desorption (TPD) profiles that were recorded following each RAIRS experiment. These experiments involve resistively heating the HOPG surface at a linear heating rate (in these experiments 0.5 K s^–1^) and monitoring the desorption products using the QMS. These desorption profiles show the relative number of molecules that were on the surface during the RAIRS experiments, which were then compared to show that the uncertainty is generally 10–20% between experiments. This uncertainty is accounted for in the analysis of the IR absorption parameters but does not have a significant impact on the errors of the results.

RAIRS experiments were performed using a Fourier transform infrared (FTIR) spectrometer (Thermo Nicolet 6700) coupled to an external liquid nitrogen cooled mercury cadmium telluride (MCT) detector. An optimal grazing angle of 15° was used to recover the maximum reflectance signal based on a calculation of the optical properties of the HOPG surface as a function of reflection angle.^36^ All spectra were recorded by coadding 256 scans at 4 cm^–1^ resolution, taking 2 min 40 s to collect each spectrum (approximately 0.63 s per scan). Background spectra were collected at 28 K before dosing to give the relative IR signal compared to a clean HOPG surface. Spectra were collected in reflectance mode (directly proportional to transmission) and then converted to absorbance for analysis. Reflectance is defined here as ΔR/R, being the difference in attenuated IR light between the clean and ice-covered HOPG surface. A more in-depth definition of the relation between transmittance, absorbance and reflectance is given in the Supporting Information.

Methyl acetate and methyl propanoate were purchased from Sigma-Aldrich at their highest available purity (≥99.5%) and then further purified through several freeze–pump–thaw cycles to remove any dissolved gases that were trapped during decanting into the sample reservoirs.

The IR absorption coefficients, cross sections, and band strengths were calculated using a modified Beer–Lambert law which equates the difference between the transmitted/reflected IR intensities of the clean HOPG surface and of the molecular ices (converted to absorbances) to the peak heights or integrated areas of each vibrational mode in the spectra.^14,15,19^ In solid-state spectroscopy, strong intermolecular interactions within the ice structure cause spectral broadening which affects the vibrational band peak heights. This must be considered when comparing spectra between corresponding gas- and solid-phase species. However, this is in fact a strength of the analysis method described here, as these intermolecular interactions are implicitly accounted for when evaluating the IR absorption parameters from experimental data of molecular ices.

**Figure 1:**
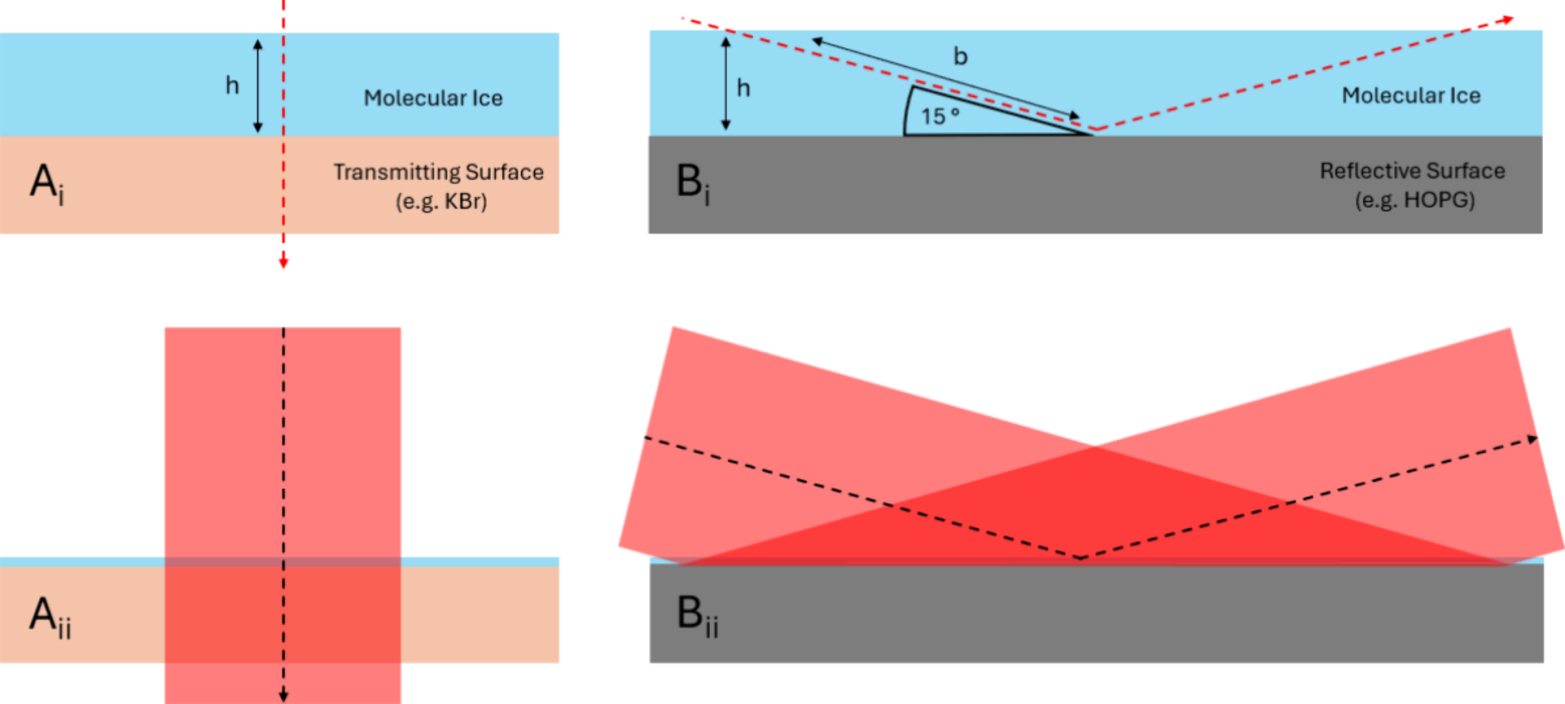
Schematic of the IR beam path length passing through a molecular ice for (A) a transmitting surface such as KBr and (B) a reflective surface such as HOPG for RAIRS experiments. For both surfaces the IR beam path is shown for (i) a point source and (ii) the ∼5 mm diameter IR beam spot used in this work.

The modified Beer–Lambert laws are presented as eqs 4, 7 and 8, and the derivations are given in the Supporting Information. The absorption coefficient, α′ is given by
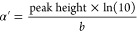
4where the peak height is taken as the maximum intensity of each vibrational band and *b* is the path length of the IR beam through the ice. The factor of ln(10) converts the absorbance to an optical depth-scale, essentially accounting for the spectrometer response. In transmission experiments, the path length of the IR beam is equal to the ice thickness (*h*), but for reflection experiments, this will depend on the beam path as it reflects at the surface. This is calculated using trigonometry, based on the known angle of incidence for our setup and the calculated ice thickness (eqs 1–3) and is shown in Figure 1B_i_.

The path length through the ice, *b,* can then be calculated using
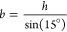
5

The full IR path length in the RAIRS experiment is equal to 2b to account for the passage of the incident and reflected beams of light through the ice.

The absorption cross sections and band strengths were calculated using the column densities (*N*_*m,c*_) according to eqs 7 and 8. The column density is a measure of how many molecules are present within a certain column of defined cross-section. In transmission experiments, the column density is equal to the surface density because the cross-section is a vertical column through the ice on the surface. However, as the path length during RAIRS experiments is given according to eq 5 and the considerations discussed above, the column density is therefore given by rearranging eq 3, and substituting the ice thickness for the angle-dependent path length, as shown in eq 6:
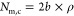
6

The absorption cross sections (σ) and band strengths (*A*^′^) were then calculated as follows:
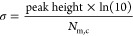
7
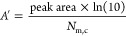
8where σ is the absorption cross-section (cm^–2^), *A*^′^ is the band strength (cm molecule^–1^), *N*_*m,c*_ is the column density (molecules cm^–2^) and the factor of ln(10) converts the absorbance to an optical depth scale.

## Results and Discussion

**Figure 2:**
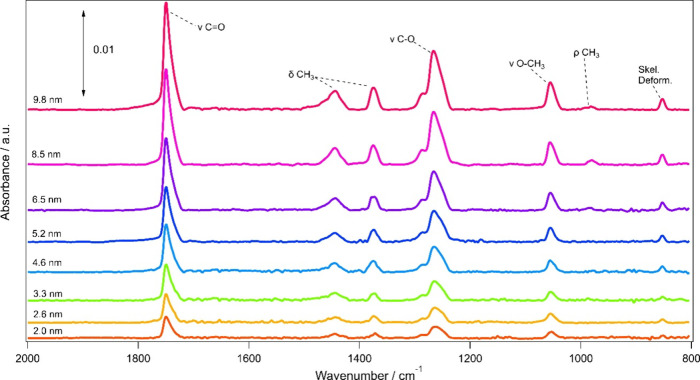
RAIR spectra for methyl acetate ices of thicknesses between 2.0 and 9.8 nm adsorbed on HOPG at 28 K.

Figure 2 shows IR absorption spectra for amorphous methyl acetate ices of increasing thickness grown on HOPG at 28 K. The IR bands increase in intensity as the exposure increases but do not saturate, implying that the ice is growing as a physisorbed multilayer.^27^ The band assignments have previously been discussed in detail elsewhere,^14,37^ but briefly from left to right the assignments are as follows: ν C = O (1754 cm^–1^), δ_*as/s*_ O–CH_3_ (1446 cm^–1^), δ_*s*_ C–CH_3_ (1377 cm^–1^), ν C–O (1270 cm^–1^), ν O–CH_3_ (1056 cm^–1^), ρ *CH*_*3*_ (982 cm^–1^), and skeletal deformation (852 cm^–1^). These assignments are also presented in Table 1. The recorded spectra also show high wavenumber bands due to the methyl C–H stretching modes at 2999 cm^–1^ (ν_as_ O/C–CH_3_) and 2958 cm^–1^ (ν_s_ O/C–CH_3_).^37^ However, as these modes have significantly lower intensity than the other bands presented, they have been omitted from the IR absorption parameter analysis and are therefore not shown in Figure 2.

**Table 1:** Infrared Absorption Coefficients for Amorphous Methyl Acetate Ices Grown at 28 K on HOPG^a^

Vibrational band assignment	Band Position/cm^–1^	α’/cm^–1^	α’ (scaled)/cm^–1^	Lit.^14^ α’/cm^–1^
ν C=O	1754	6973 ± 460	19106 ± 1260	13600
δ_*as*_ (O)CH_3_ + δ_*s*_ (O)CH_3_	1446	1071 ± 173	2935 ± 474	2980
δ_*s*_ (C)CH_3_	1377	1363 ± 223	3734 ± 611	3840
ν C(O)-O	1270	3706 ± 273	10153 ± 748	10000
ν O–CH_3_	1056	1588 ± 239	4351 ± 656	4900
Skel. Def.	852	698 ± 132	1913 ± 363	2240

a The scaled values are produced by correcting for the metal-surface selection rule (MSSR) and metal-surface enhancement factor (MSEF), as discussed in the main text.

**Figure 3:**
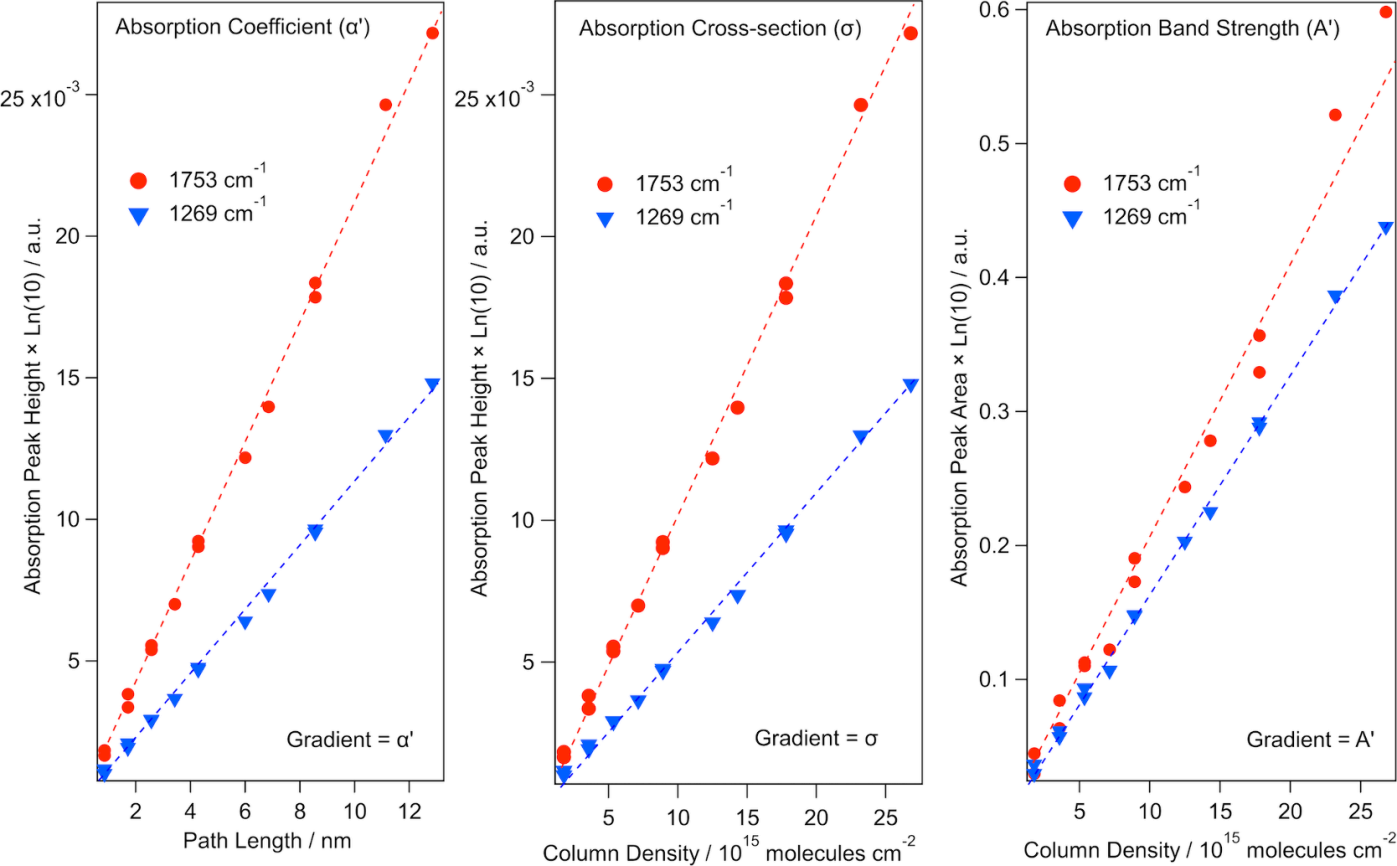
Plots for the determination of α’, σ, and A’ for the 1753 and 1269 cm^–1^ vibrational modes of amorphous methyl acetate.

From these IR data, the IR absorption parameters for methyl acetate were then calculated according to eqs 4, 7and 8 by plotting either the IR band peak height (for α’ and σ) or peak area (for A’) against the column density (for σ or A’) or path length (for α’) to give the absorption parameters as the gradient. Example plots for the determination of α’, σ and A’ for selected bands (1753 and 1269 cm^–1^) of amorphous methyl acetate are show in Figure 3. The remaining plots are shown in the Supporting Information as Figures S1–S6. The calculated absorption parameters determined from these plots are presented in Tables 1–3.

**Table 2:** Infrared Absorption Cross Sections for Amorphous Methyl Acetate Ices Grown at 28 K on HOPG^a^

Vibrational Assignment	Band Position/cm^–1^	σ/10^–18^ cm^2^	σ (scaled)/10^–18^ cm^2^
ν C=O	1754	1.03 ± 0.07	2.82 ± 0.19
δ_*as*_ (O)CH_3_ + δ_*s*_ (O)CH_3_	1446	0.16 ± 0.03	0.43 ± 0.07
δ_*s*_ (C)CH_3_	1377	0.20 ± 0.03	0.55 ± 0.09
ν C(O)-O	1270	0.55 ± 0.04	1.50 ± 0.11
ν O–CH_3_	1056	0.23 ± 0.04	0.64 ± 0.10
Skel. Def.	852	0.10 ± 0.02	0.28 ± 0.05

a The scaled values are produced by correcting for the MSSR and MSEF, as discussed in the main text.

**Table 3:** Infrared Absorption Band Strengths for Amorphous Methyl Acetate Ices Grown at 28 K on HOPG^a^

Vibrational Assignment	Integration Range/cm^–1^	A’/10^–18^ cm molec^–1^	A’ (scaled)/10^–18^ cm molec^–1^	Lit.^14^ A’/10^–18^ cm molec^–1^
ν C=O	1718−1800	21.68 ± 3.89	59.40 ± 10.66	48.0
δ_*as*_ (O)CH_3_ + δ_*s*_ (O)CH_3_	1416−1520	4.32 ± 0.80	11.83 ± 2.18	13.7
δ_*s*_ (C)CH_3_	1354−1414	3.28 ± 0.70	8.98 ± 1.92	9.21
ν C(O)-O	1230−1323	16.31 ± 0.92	44.68 ± 2.52	47.9
ν O–CH_3_	1034−1078	4.05 ± 0.62	11.11 ± 1.70	13.0
Skel. Def.	841−866	1.03 ± 0.23	2.83 ± 0.63	3.48

a The scaled values are produced by correcting for the MSSR and MSEF, as discussed in the main text.

**Figure 4:**
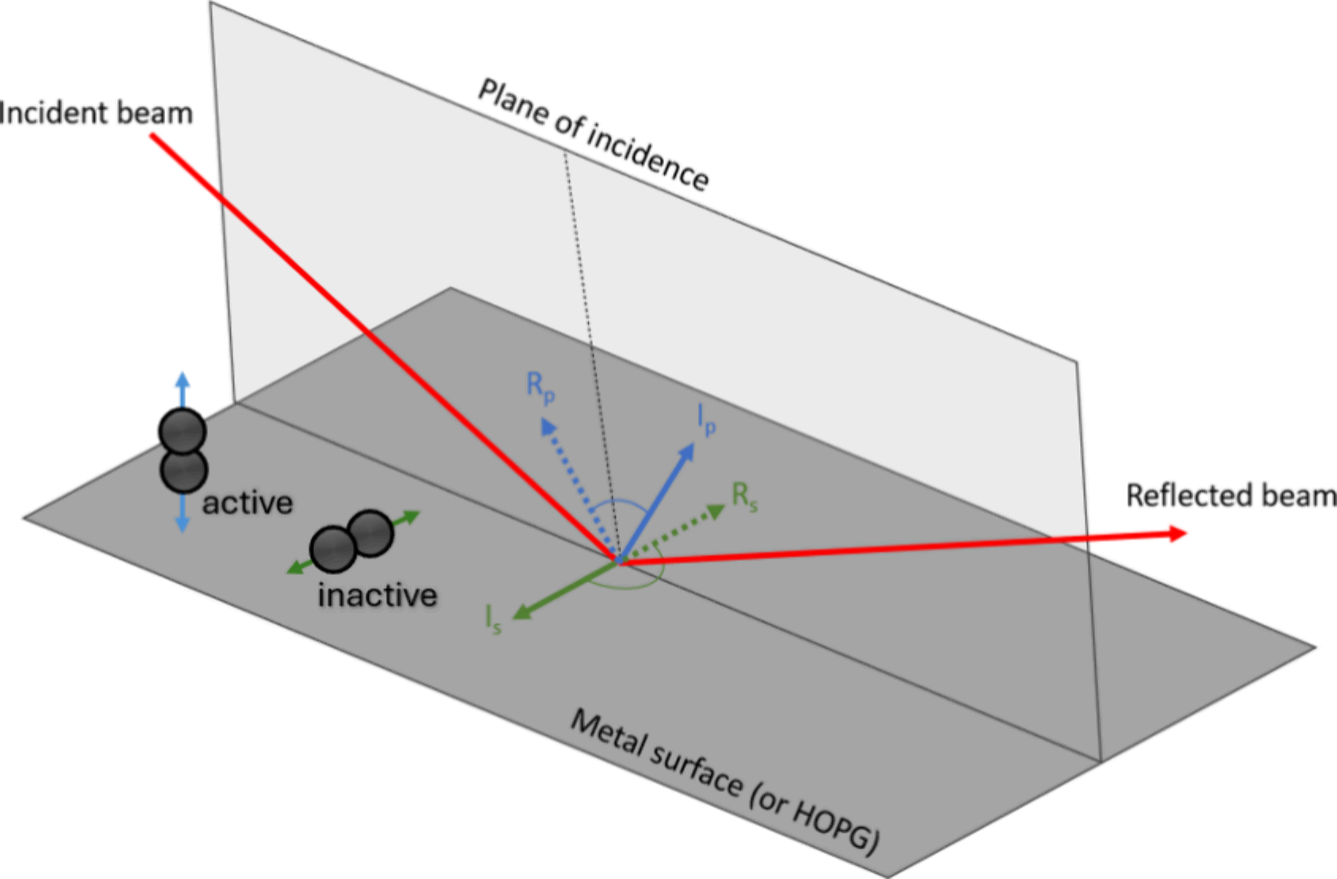
Different behavior of the *s-* and *p-*polarized light components of the IR beam when reflecting at a metal or semimetal surface. *S-*polarized light is parallel to the surface and *p-*polarized light is perpendicular to the surface plane.

The IR absorption parameters presented do not initially compare well to the literature values presented in Tables 1 and 3. However, there are a few important considerations when comparing the RAIR spectra of adsorbed molecules on a metal or semimetal surface to those obtained from transmission experiments. First, we must consider the metal-surface selection rule (MSSR), and specifically how this dictates which molecular vibrations are detected using RAIRS. Although not a metal, the delocalized π electron cloud of HOPG makes it a semimetal and the material therefore obeys the MSSR. The MSSR states that only components of the dynamic dipole moment perpendicular to the surface are infrared active, while parallel components are suppressed.^24,25,36^Figure 4 demonstrates this rule by showing the electric field vectors of the incident and reflected light. The beam can be separated into two components, the s- and p-polarized light, which interact with the surface differently. The s-polarized component (seen in green in Figure 4) is parallel to the surface and undergoes a 180° shift upon reflection. Hence, at the surface, the incident and reflected beams undergo destructive interference, effectively rendering this component IR inactive. The p-polarized light (seen in blue in Figure 4) is perpendicular to the surface, and its phase shift upon reflection is dependent on the angle of incidence. The optimal angles of incidence for the IR beam on metal and HOPG surfaces are 88° and 73° respectively.^24,25,36^

Because of the MSSR, only the perpendicular components of molecular vibrations are observed with RAIRS. Assuming a totally random orientation of all molecules that have frozen on the HOPG surface, every molecule has an equal chance of laying parallel or perpendicular to the surface, indicating that during RAIRS experiments, approximately half of the molecular vibrations are inactive. To recover this lost absorption intensity, the absorbance values were multiplied by a factor of 2. It is important to note that this would not be valid for crystalline ices which have a distinct ordering of the molecules in the ice. In these cases, a proportionality factor could be applied to account for the known crystal geometry.

This approximation is supported by previous studies such as that of Lasne et al., in which both the perpendicular and parallel dipoles of amorphous N_2_O on a copper surface were observed in almost equal number using RAIRS.^38^ This was achieved by creating a rough silica underlayer that bypasses the MSSR, allowing the parallel dipole components to be observed which were hidden from the traditional RAIRS on the bare copper surface.^38^ Hence, for the amorphous ices discussed in this work, this factor of 2 is a reasonable correction based on the nature of amorphous ices deposited at cryogenic temperatures.

The second consideration is the metal-surface enhancement factor (MSEF), which describes the influence of the electric field of the metal or semimetal surface on the molecular vibrations of the adsorbed species.^39−41^ This electric field arises from localized surface plasmon resonances and has the effect of increasing the probability of the adsorbed molecules interacting with the IR light, thus increasing the absorption signal.^42^ For HOPG, this effect arises due to graphite’s delocalized π electron cloud. The exact magnitude of the MSEF will vary based on factors including surface nanostructure, local temperature, and the nature of the vibrational modes in question.^41,42^ By comparing the α’ and A’ values for adsorption on our HOPG sample in this work to those reported by Hudson et al. and shown in Tables 1, 3, 4, and 6, an approximate MSEF for reflection-to-transmission (RtT) conversion is found to be 1.37.

**Table 4:** Infrared Absorption Coefficients for Amorphous Methyl Propanoate Ices Grown at 28 K on HOPG^a^

Vibrational Assignment	Band Position/cm^–1^	α’/cm^–1^	α’ (scaled)/cm^–1^	Lit.^14^ α’/cm^–1^
ν C=O	1747	6045 ± 826	16562 ± 2264	9808
δ *(C)CH*_*3*_*+* ω (O)CH_3_ or ρ (O)CH_3_	1444	780 ± 306	2136 ± 839	1767
ω [CH_3_CH_2_] or ω (C)CH_3_	1363	1294 ± 306	3545 ± 837	3412
ρ [CH_3_CH_2_] or ν (O)C–O	1213	3429 ± 484	9394 ± 1325	7595
ω CH_3_ or ρ (C)CH_3_	1092	396 ± 177	1085 ± 484	1368
ω *CH*_*2*_ *+* ν O–CH_3_	1022	612 ± 183	1677 ± 501	1334
ω CH_3_ or ρ (C)CH_3_	970	337 ± 196	925 ± 537	639
ν C–C(O) + ν C(O)-O	854	579 ± 341	1587 ± 935	1340

a The scaled values are produced by correcting for the MSSR and MSEF, as discussed in the main text.

**Table 5:** Infrared Absorption Cross Sections for Amorphous Methyl Propanoate Ices Grown at 28 K on HOPG^a^

Vibrational Assignment	Band position/cm^–1^	σ/10^–18^ cm^2^	σ (scaled)/10^–18^ cm^2^
ν C=O	1747	1.26 ± 0.17	3.45 ± 0.47
δ *(C)CH*_*3*_*+* ω (O)CH_3_ or ρ (O)CH_3_	1444	0.16 ± 0.06	0.44 ± 0.17
ω [CH_3_CH_2_] or ω (C)CH_3_	1363	0.27 ± 0.06	0.74 ± 0.17
ρ [CH_3_CH_2_] or ν (O)C–O	1213	0.71 ± 0.10	1.96 ± 0.28
ω CH_3_ or ρ (C)CH_3_	1092	0.08 ± 0.04	0.23 ± 0.10
ω *CH*_*2*_ *+* ν O–CH_3_	1022	0.13 ± 0.04	0.35 ± 0.10
ω CH_3_ or ρ (C)CH_3_	970	0.07 ± 0.04	0.19 ± 0.11
ν C–C(O) + ν C(O)-O	854	0.12 ± 0.07	0.33 ± 0.19

a The scaled values are produced by correcting for the MSSR and MSEF, as discussed in the main text.

**Table 6:** Infrared Absorption Band Strengths for Amorphous Methyl Propanoate Ices Grown at 28 K on HOPG^a^

Vibrational Assignment	Integration Range/cm^–1^	A’/10^–18^ cm molec^–1^	A’ (scaled)/10^–18^ cm molec^–1^	Lit.^14^ A’/10^–18^ cm molec^–1^
ν C=O	1718−1767	18.07 ± 2.70	49.51 ± 7.39	41.8
δ *(C)CH*_*3*_ *+* ω (O)CH_3_ or ρ (O)CH_3_	1421−1481	5.86 ± 2.86	16.06 ± 7.84	15.3
ω [CH_3_CH_2_] or ω (C)CH_3_	1344−1379	3.37 ± 0.77	9.23 ± 2.11	11.4
ρ [CH_3_CH_2_] or ν (O)C–O	1194−1238	12.93 ± 2.53	35.43 ± 6.93	51.0
ω CH_3_ or ρ (C)CH_3_	1082−1102	0.78 ± 0.24	2.13 ± 0.64	4.0
ω *CH*_*2*_ *+* ν O–CH_3_	1082−1194	1.55 ± 0.74	4.25 ± 2.04	3.4
ω CH_3_ or ρ (C)CH_3_	953−987	1.10 ± 0.62	3.02 ± 1.69	1.7
ν C–C(O) + ν C(O)-O	839−866	1.09 ± 0.80	2.98 ± 2.19	2.5

a The scaled values are produced by correcting for the MSSR and MSEF, as discussed in the main text.

The reflection of light in a RAIRS experiment at a metal surface was originally described by Greenler.^24^ In this work, a three-layer (vacuum, thin-film and metal-surface) model of IR light reflecting on a surface was evaluated, demonstrating the effects of the MSSR, both in the suppression of the parallel dipole components and the enhancement of the perpendicular components.^24^ This approach was later used for graphite surfaces in the work of Heidberg et al., from which a graphite surface sensitivity factor can be retrieved from the angle-dependent square of the electric field strength at the surface.^36^ By evaluating this same model for our experimental setup (a grazing angle of 15° on HOPG), an expected RtT conversion factor for the perpendicular dipole components is ≈1.9, which compares well to our experimental value of 1.37.

The only other comparison available in the literature is the transmission-to-reflection (TtR) conversion factor reported by Santos et al. for a gold-plated copper surface of 3.2.^29,30^ In that work, they report this factor as a direct conversion determined using laser interferometry techniques. Comparing the two conversion factors is therefore difficult since the origin of the conversion factor given by Santos et al. is not clear. However, if this factor includes contributions from the MSSR and MSEF in a similar way as described here, and that the conversion factor is equal, but opposite, for RtT and TtR, then their reported factor of 3.2 for a metal surface can be compared to our factor of 0.73 for HOPG (converted from RtT to TtR). This smaller factor is expected, since HOPG is known to have a weaker surface electric field, and therefore weaker MSEF, than a metal surface.^40,41^

**Figure 5:**
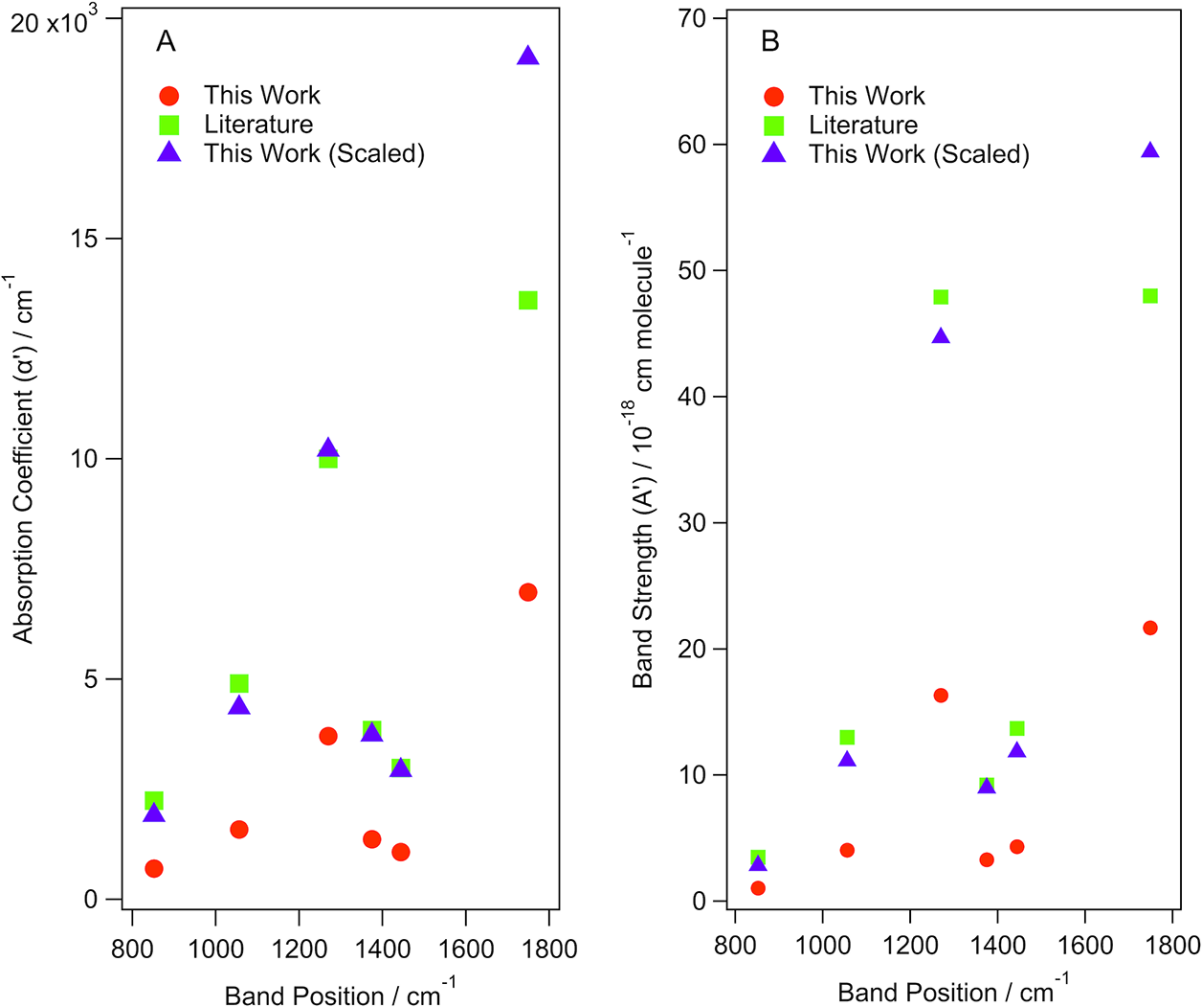
Comparison of the (A) absorption coefficients and (B) band strengths determined in this work for amorphous methyl acetate before and after applying the factors to account for the MSSR and MSEF. The literature values from Hudson et al. are also shown for reference.^14^

The RtT factor determined here for HOPG was then applied to the α’, σ, and A’ values in Tables 1–3. Figure 5 shows how the scaled and nonscaled values compare to literature.

**Figure 6:**
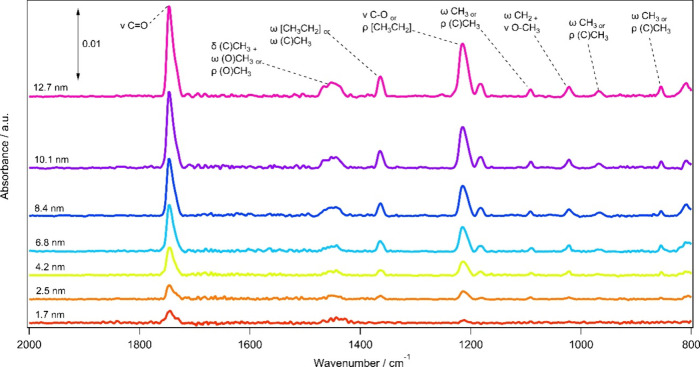
RAIR spectra for methyl propanoate ices of thicknesses between 1.7 and 12.7 nm adsorbed on HOPG at 28 K.

Having demonstrated the viability of this method, the experiments and analysis were repeated for another simple ester, methyl propanoate, which has relevance as a potential candidate for detection in the ISM.^16^Figure 6 shows IR absorption data for amorphous methyl propanoate ices of increasing thickness grown on HOPG at 28 K. This species behaves extremely similarly to methyl acetate, as discussed in detail previously.^37^ The approximate band assignments, from left to right, are ν C = O (1747 cm^–1^), δ *(C)CH*_*3*_*+* ω (O)CH_3_ or ρ (O)CH_3_ (1444 cm^–1^), ω [CH_3_CH_2_] or ω (C)CH_3_ (1363 cm^–1^), ρ [CH_3_CH_2_] or ν (O)C–O (1213 cm^–1^), ω CH_3_ or ρ (C)CH_3_ (1092 cm^–1^), ω *CH*_*2*_*+* ν O–CH_3_ (1022 cm^–1^), ω CH_3_ or ρ (C)CH_3_ (972 cm^–1^) and ν C–C(O) + ν C(O)-O (854 cm^–1^). Again, the recorded spectra show the high wavenumber bands belonging to the methyl C–H stretching modes at 2983 and 2956 cm^–1^ (ν_as_ / ν_s_ (O)CH_3_ + ν_as_ / ν_s_ (C)CH_3_ + ν_as_ / ν_s_ CH_2_).^16,37^ As for methyl acetate, these have been omitted due to low intensity which would introduce too much error.

Following the same analysis as before, the IR absorption coefficients, cross sections and band strengths were calculated for methyl propanoate and are presented in Tables 4–6.

**Figure 7:**
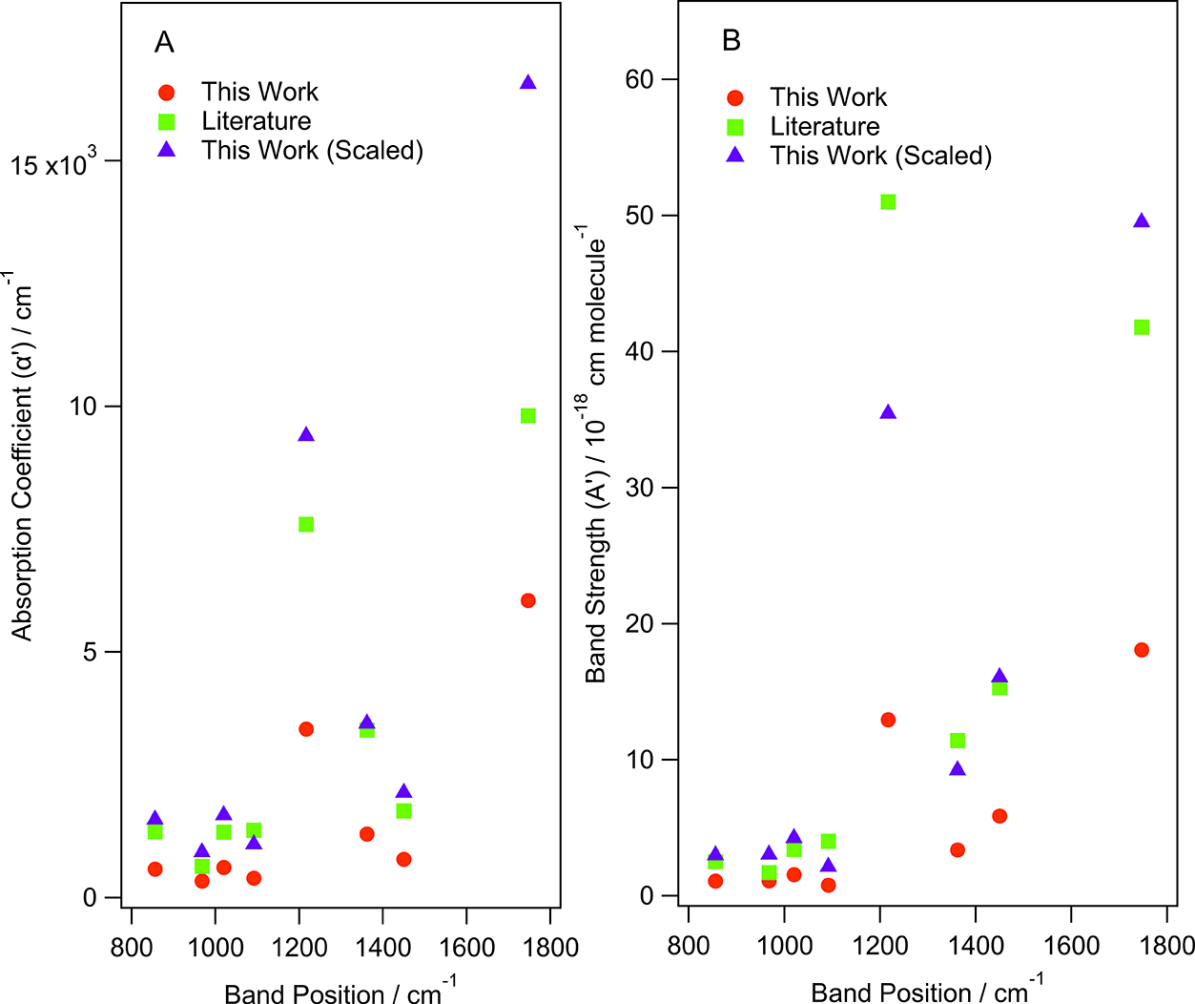
Comparison of the (A) absorption coefficients and (B) band strengths for amorphous methyl propanoate determined in this work before and after applying the factors to account for the MSSR and MSEF. The literature values from Hudson et al. are also shown for reference.^14^

The same RtT conversion was applied to the α', σ, and A’ values in Tables 4–6. These scaled values were then compared to the literature values reported by Hudson et al.,^14,16^ with Figure 7 showing how the scaled and nonscaled values compare to literature.

The values that differ more significantly when compared to the literature generally involve the more intense vibrations which include the oxygens of the ester species. This is most likely a consequence of our assumption for correcting the MSSR, in which all adsorbed molecules have an equal chance of laying parallel or perpendicular to the surface. From previous studies, spontaneous dipole orientation effects have been shown to influence the initial orientations of adsorbates, and so the more electronegative oxygen atoms in the methyl acetate and methyl propanoate molecules could be influencing the initial geometries, even in the amorphous ices.^43,44^ The influence of these spontaneous dipole orientation effects on the vibrational energies is surface independent, and so would be observed for both transmission and reflection IR experiments. However, for RAIRS experiments it would potentially affect the number of observed dipoles due to the MSSR.

## Conclusions

This work demonstrates the applicability of existing methods for calculating IR absorption coefficients, cross sections, and band strengths when applied to RAIRS data of molecular ices deposited on a cryogenically cooled graphite surface. The results show systematic deviations when compared to transmission spectroscopy methods, which can be attributed to the metal-surface selection rule (MSSR) and the metal-surface enhancement factor (MSEF). These deviations can be corrected using a derived conversion factor.

The MSSR, which limits vibrational modes detectable in RAIRS to those with dipole changes with a component perpendicular to the surface, required a correction factor of 2 to account for lost intensity from parallel vibrational modes. The MSEF, which quantifies the amplification of absorption signals due to the delocalized π-electron field of HOPG, was determined to give a reflection-to-transmission (RtT) conversion factor of approximately 1.37. This value is in line with the only available literature comparison, the transmission-to-reflection (TtR) conversion factor reported by Santos et al. and demonstrates the weaker enhancement of absorption for adsorbates bound to HOPG compared to metals.^29,30^

After applying these corrections, the RAIRS-derived absorption parameters closely match literature values obtained from transmission spectroscopy, validating the methodology for amorphous ices. However, potential spontaneous dipole orientation effects may cause a breakdown in the assumption for correcting for the MSSR, with highly electronegative vibrational modes possibly having an orientational preference when adsorbing, even at cryogenic temperatures. These results highlight RAIRS as a robust technique for deriving spectroscopic parameters under experimental constraints where traditional transmission methods are not feasible.

This methodology is subject to the same limitations as any RAIRS experiments, in that ices must be thin enough for the MSSR to be valid.^24,25^ As also described, any factors that affect the orientational distribution of the ices (i.e., thermal or energetic processing, spontaneous dipole effects, crystallization etc.) will also affect the accuracy of the absorption parameters derived.

With the IR absorption parameters discussed in this work being important in both observational and theoretical astrochemistry, we have demonstrated how IR data collected using multiple different experimental methods can all be used to contribute to astrochemical research moving forward.

## Data Availability

Raw experimental data and computational output files are available and can be found at the University of Sussex data repository; at 10.25377/sussex.26058322.

## Supporting Information Available

Discussion of how infrared transmittance, reflectance, and absorbance; derivations of the modified Beer–Lambert laws employed in this work; and additional figures for the determination of α’, σ, and A’ (PDF)

## Dedication

This paper is dedicated to the memory of Professor Harold Linnartz. This work was examined as part of the PhD thesis of the first author by Professor Linnartz in December 2023.
