# Supplementary material for: Evaluating Infrared Absorption Parameters for Low-Temperature Ices Using Reflection–Absorption Infrared Spectroscopy
Source: ACS Earth Space Chem. 2025 Mar 4;9(3):746–56. doi: 10.1021/acsearthspacechem.4c00394 (PMC11931534; doi:10.1021/acsearthspacechem.4c00394)
Supplement: Supplementary file 1 — sp4c00394_si_001.pdf [file sp4c00394_si_001.pdf]

Evaluating Infrared Absorption Parameters for Low Temperature Ices using Reflection-Absorption Infrared Spectroscopy – Supplementary Information

Jack E. Fulker[1], Martin McCoustra[2], and Wendy A. Brown[1]*

1: *Department of Chemistry, University of Sussex, Falmer, BRIGHTON, BN1 9QJ, UK*

2: *Institute of Chemical Sciences, Heriot-Watt University, EDINBURGH, EH14 4AS, UK*

Defining infrared transmittance, reflectance and absorbance

Let us assume that I_0 and I_{trans} are the incident and transmitted intensities in an absorption/transmission experiment. In Napierian terms, we can relate these through

$$I_{trans} = I_0 e^{-\sigma\rho d} \tag{S1}$$

where σ is the absorption cross-section, ρ is the number density of the absorbing medium (sometimes given as n) and d is the optical path length (sometimes given as l). The latter can also be combined to give the column density, N_m (=ρd).

Alternatively, we can represent this in Decadic terms and link it to the well-known Beer-Lambert Law

$$A = \varepsilon c d \tag{S2}$$

where A is the absorbance, ε is the extinction coefficient, and c is the molar concentration of the absorbing medium. By combining S1 and S2, we arrive at:

$$I_{trans} = I_0 10^{-\varepsilon c d} = I_0 10^{-A} \;\rightarrow\; I_{trans} = I_0 e^{-A\ln(10)} \tag{S3}$$

and

$$\ln\left(\frac{I_{trans}}{I_0}\right) = -A\ln(10) = -\sigma\rho d = -\sigma N_m \tag{S4}$$

From which we obtain the relationship

$$A = \frac{\sigma\rho d}{\ln(10)} = \frac{\sigma N_m}{\ln(10)} \tag{S5}$$

Infrared spectra recorded in transmission and reflection modes are related to each other as follows:

$$\frac{I_{trans}}{I_0} \propto \frac{\Delta R}{R_0} = \frac{R_0 - R_s}{R_0} \qquad \text{(S6)}$$

where R_0 is the 'background' reflected intensity (from the clean surface) and R_s is the signal-bearing reflected intensity (from the ice-covered surface).

Deriving the modified Beer-Lambert Laws

For our experimental RAIRS data, in which reflectance has been converted to absorbance using equations S3-6, we can intuitively equate *A* to the absorption band peak heights.

Equation S5 therefore becomes the modified Beer-Lambert Law for calculating the absorption cross-section (σ).

$$\ln(10) peak\ height = \sigma \times N_m \ \rightarrow \ \sigma = \frac{peak\ height \times ln(10)}{N_m} \qquad \text{(S7)}$$

By defining an absorption band strength as the integrated absorption cross-section over an entire vibrational band, we arrive at the modified Beer-Lambert Law for calculating the absorption band strengths (A').

$$\ln(10) peak\ area = A' \times N_m \rightarrow \ A' = \frac{peak\ area \times ln(10)}{N_m} \qquad \text{(S8)}$$

Finally, by defining the absorption coefficient as

$$\alpha' = \sigma\rho \qquad \text{(S9)}$$

we arrive at the modified Beer-Lambert Law for calculating the absorption coefficient (α').

$$\ln(10) peak\ height = \alpha' \times path\ length \ \rightarrow \ \alpha' = \frac{peak\ height \times ln(10)}{path\ length} \qquad \text{(S10)}$$

Supplementary Figures

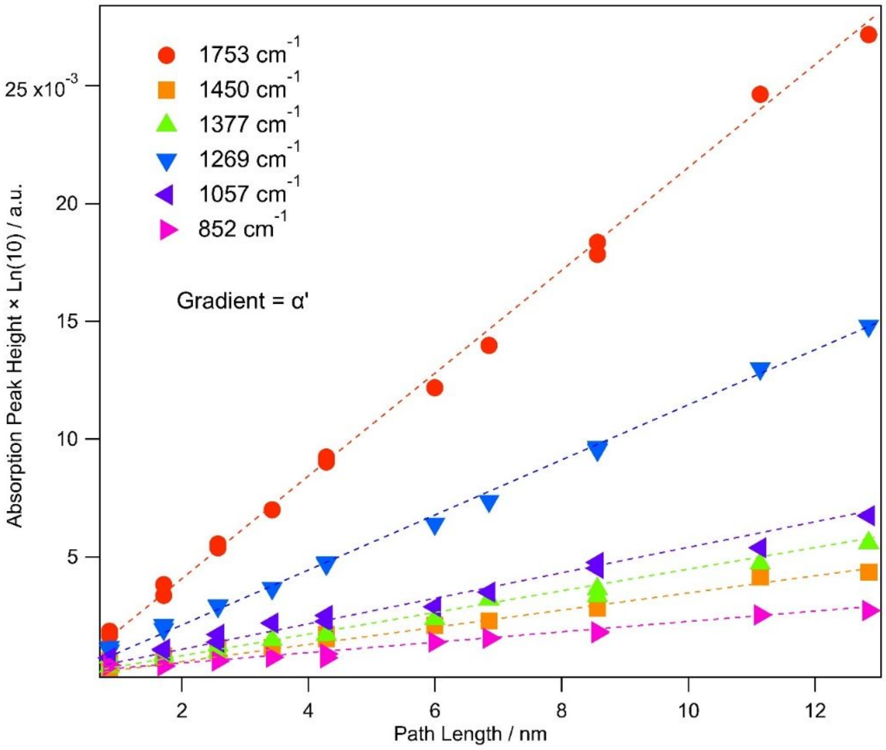

Figure S1: Plots of absorbance peak height against path length that allow the derivation of the absorption coefficients for amorphous methyl acetate. Peak heights are taken from the RAIRS data for ices of thicknesses between 3.3 – 9.8 nm adsorbed on HOPG at 28 K (Figure 2).

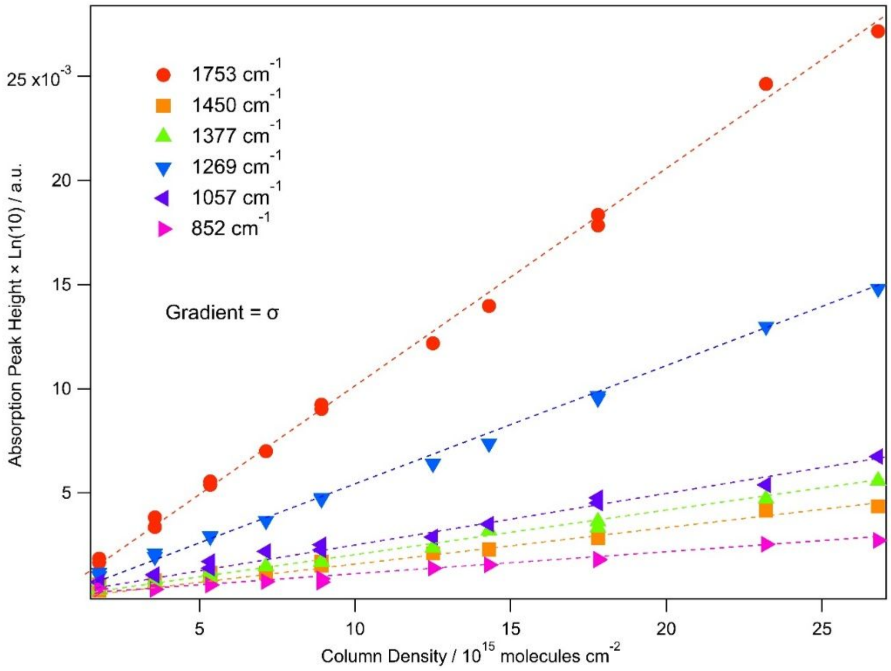

Figure S2: Plots of absorbance peak height against column density that allow the derivation of the absorption cross-sections for amorphous methyl acetate. Peak heights are taken from the RAIRS data for ices of thicknesses between 3.3 – 9.8 nm adsorbed on HOPG at 28 K (Figure 2).

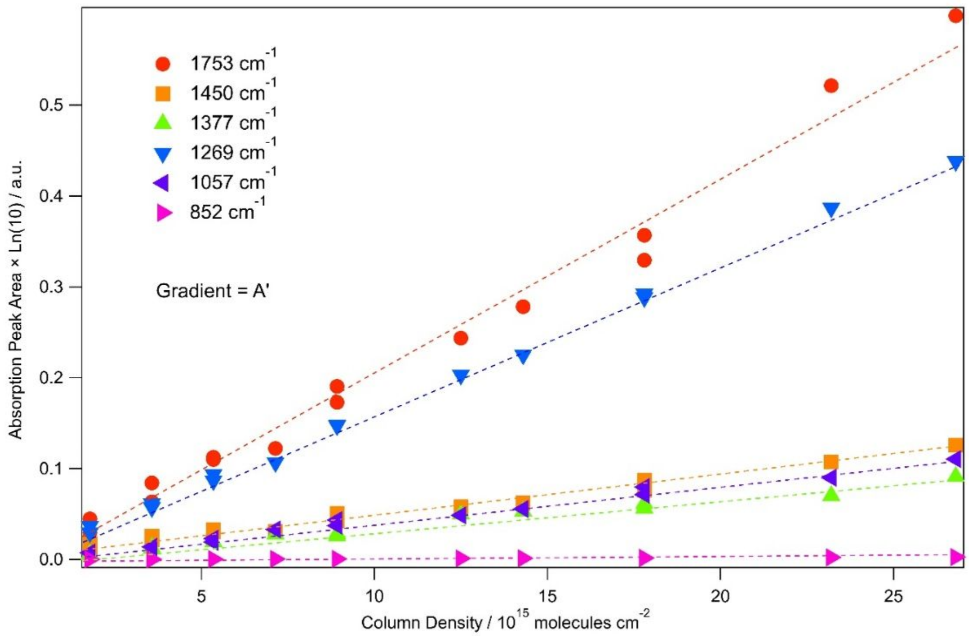

Figure S3: Plots of integrated absorbance peak area against column density that allow the derivation of the absorption band strengths for amorphous methyl acetate. Integrated absorbance peak areas are taken from the RAIRS data for ices of thicknesses between 3.3 – 9.8 nm adsorbed on HOPG at 28 K (Figure 2).

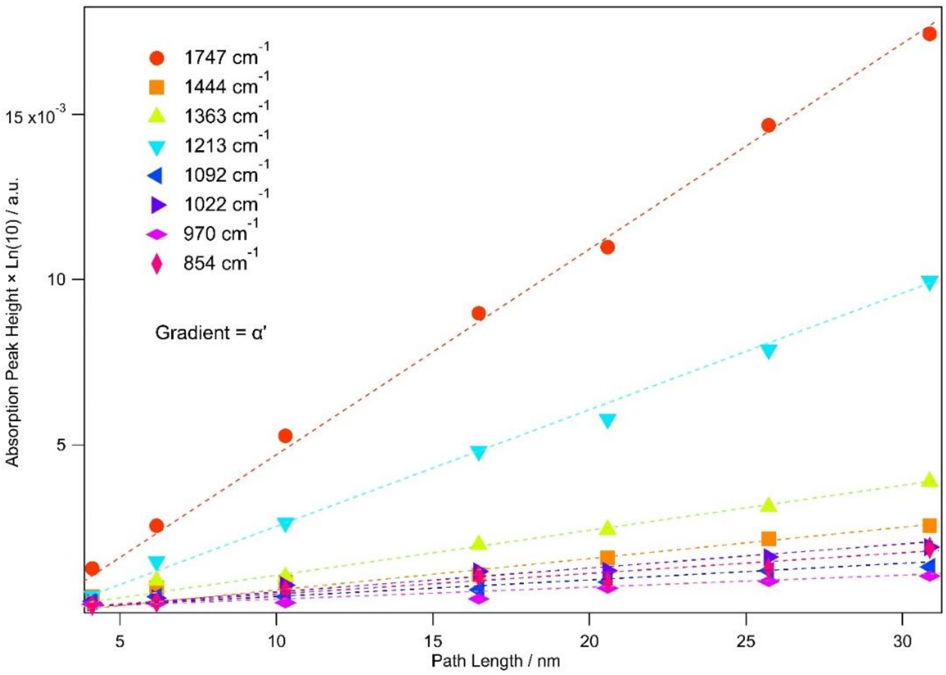

Figure S4: Plots of absorbance peak height against path length that allow the derivation of the absorption coefficients for amorphous methyl propanoate. Peak heights are taken from the RAIRS data for ices of thicknesses between 1.7 – 12.7 nm adsorbed on HOPG at 28 K (Figure 6).

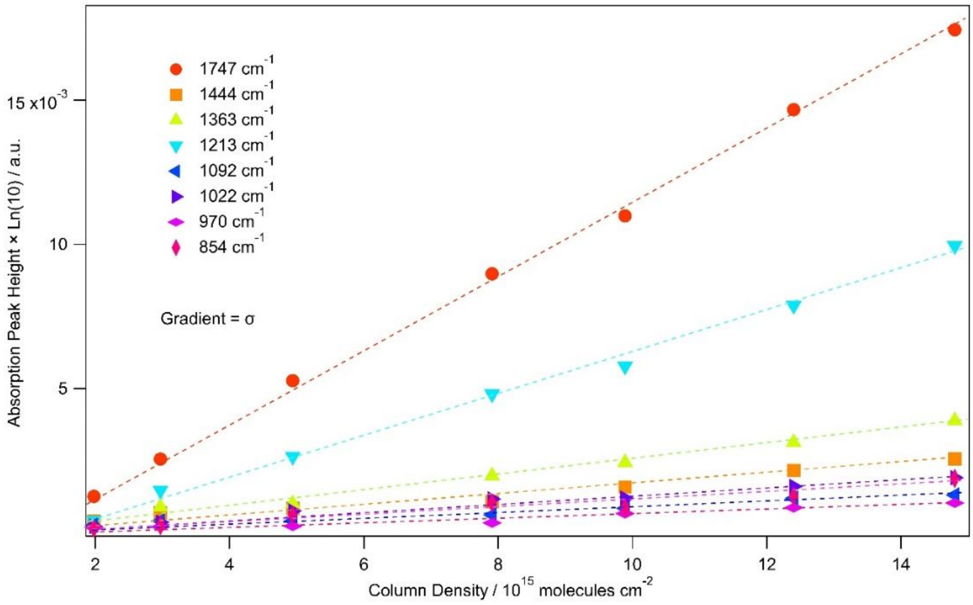

Figure S5: Plots of absorbance peak height against column density that allow the derivation of the absorption cross-sections for amorphous methyl propanoate. Peak heights are taken from the RAIRS data for ices of thicknesses between 1.7 – 12.7 nm adsorbed on HOPG at 28 K (Figure 6).

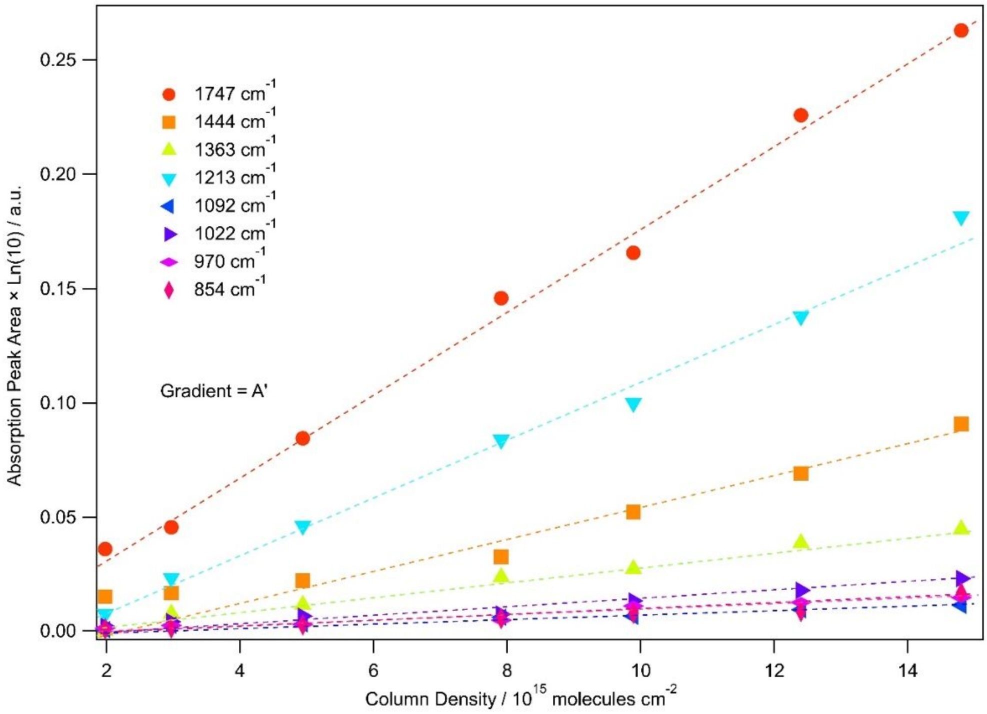

Figure S6: Plots of integrated absorbance peak area against column density that allow the derivation of the absorption band strengths for amorphous methyl propanoate. Integrated absorbance peak areas are taken from the RAIRS data for ices of thicknesses between 1.7 – 12.7 nm adsorbed on HOPG at 28 K (Figure 6).
